# Tibialis Posterior Transfer for Foot Drop: The Difference in Outcome for Two Different Attachment Sites

**DOI:** 10.7759/cureus.18461

**Published:** 2021-10-03

**Authors:** Muhammad Imran Khan, Owais Ahmed, Sobia Yasmeen, Rabah Saadique, Mirza Shehab A Beg

**Affiliations:** 1 Plastic and Reconstructive Surgery, Liaquat National Hospital and Medical College, Karachi, PAK

**Keywords:** interosseous route, 2nd metatarsal, tibialis posterior tendon, tibialis anterior tend, tendon transfer, foot drop

## Abstract

Introduction

Common peroneal nerve injury leading to foot drop is of multifactorial etiology. The goal is to restore a normal toe-heel gait. Various static or dynamic surgical options are being performed. Among all, tendon transfer is the most commonly performed procedure with its different dorsal attachment sites on the foot i.e. tendon to bone or tendon to tendon transfer. The objective of our study was to evaluate the outcomes of two methods of transfer in terms of attachments sites on functional outcomes.

Materials and methods

In a retrospective study conducted at Liaquat National Hospital Karachi, a total of 38 patients were included. All of them were operated upon for foot drop from June 2015 to May 2018. A total of 32 patients showed up for the follow-up, 17 patients underwent tibialis posterior transfer with attachment on the second metatarsal and 15 on the tibialis anterior tendon. Functional outcome was assessed by grading of active foot dorsiflexion at six months and at the time of the study by and categorized as excellent, good, moderate, and poor.

Results

Most of the patients in both groups were male, and the mechanism of injury was penetrating trauma. At six months post-operatively, the majority of the patients in both groups showed excellent to good category of active dorsiflexion. At the time of the study (mean 34.4 months postoperatively) patients with insertion at second metatarsal were found to have active dorsiflexion as: excellent: 6 (35.3%), good: 8 (47.1%), moderate: 3 (17.6%), and for insertion at Tibialis Anterior tendon: excellent: 1 (6.7%), good: 6 (40.0%), moderate: 6 (40.0%) and poor: 2 (6.2%). These results were compared using the chi-square test and it was found to be statistically significant (p-value: 0.016).

Conclusion

Insertion at second metatarsal gives more favorable results as compared to insertion at tibialis anterior with balanced dorsiflexion.

## Introduction

The common peroneal nerve is the most frequently affected peripheral nerve of the lower extremity leading to foot drop, is of varied etiology [1]. The common cause is trauma [2]. It results in loss of dorsiflexion, ankle eversion, and toes extension. The equinovarus deformity may develop with intact tibialis posterior [3]. The success rate of nerve repair has increased with recent advances in microsurgery but still, a large number of injuries lead to permanent foot drop [1].

During normal walking, when the heel strikes the ground, the ankle remains in either a slight extension or a neutral position, and during the swing phase, an active extension of the toes and ankle is required to clear the ground [2]. In foot drop during heel strike, the patient slaps the foot on the ground and in the swing phase, drags it along the ground [3]. The patient has to flexes the hip more than normal to lift the entire foot off the ground i.e. high stepping gait [2]. Some patients use ankle-foot orthoses (AFO) that prevent plantar flexion more than neutral [2]. 

The objective of reconstruction is to restore normal toe-heel gait [1,4]. Various static or dynamic surgical options are available to correct this deformity including tenodesis, arthrodesis, and tendon transfers [5]. Dynamic tendon transfers are considered the gold standard [6]. It restores the dorsiflexion of the foot and allows near-normal functional activity, and also prevents the equinovarus deformity caused by the TP tendon [7]. The route of transfer (circumtibial versus interosseous) and fixation site at the foot dorsum (tendon-to-bone or tendon-to-tendon) is still debatable in the literature [1, 6].

We used two different attachment sites in the foot, one is tendon-to-bone fixation i.e. looping the tibialis posterior tendon around the second metatarsal (a modification of classic Barr’s procedure) [8], and the second one to the tibialis anterior tendon. The objective of our study was to evaluate the functional outcomes of these two methods of tendon transfer by using the criteria described by Carayon et al. [9].

## Materials and methods

It was a retrospective study conducted at the department of plastics and reconstruction surgery, Liaquat National Hospital. A search of patient's records from hospital information management system (HIMS) and outpatient registry was done and extracted the data of 42 patients irrespective of gender or age. All the patients were operated upon for foot drop secondary to peroneal nerve injury, and tibialis posterior tendon was transferred to foot dorsum from June 2015 to May 2018. For homogeneity of the study, the patients presented with permanent foot drop after peroneal nerve injury without soft tissue loss or concomitant tendon or bone injury of the same limb treated with tibialis posterior tendon transfer to the foot dorsum using interosseous route were included. The distal fixation site of the tibialis posterior (TP) tendon was tendon to bone (loop around 2nd metatarsal) or tendon-to-tendon (tibialis anterior - TA). The reason for choosing a specific distal fixation site was the surgeon’s preference. Patients with excessive crush injury, extensive soft tissue loss, and multiple nerve injuries, with underlying bone disease or deformity, prior history of vascular or nerve injury of the affected limb, or with known vascular/ neurological disorder were excluded from the study.

**Figure 1:**
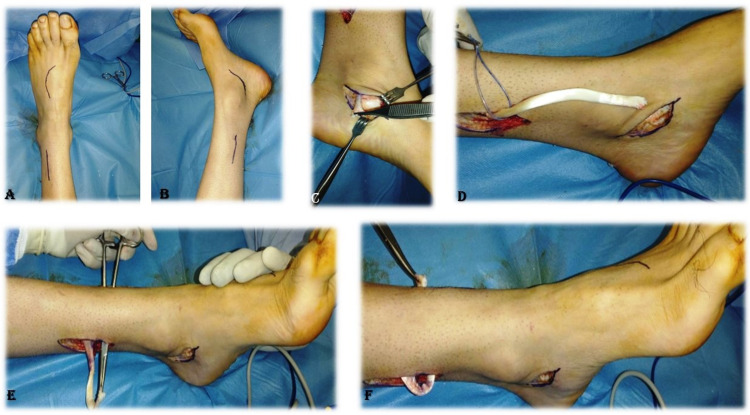
Marking, identification, division and tunneling of tibialis posterior tendon through interosseous membrane. Marking (A, B), identification of tibialis posterior tendon (C). Division and proximal delivery of tibialis posterior tendon (D). Tunneling of tibialis posterior tendon through the interosseous membrane (E, F).

**Figure 2:**
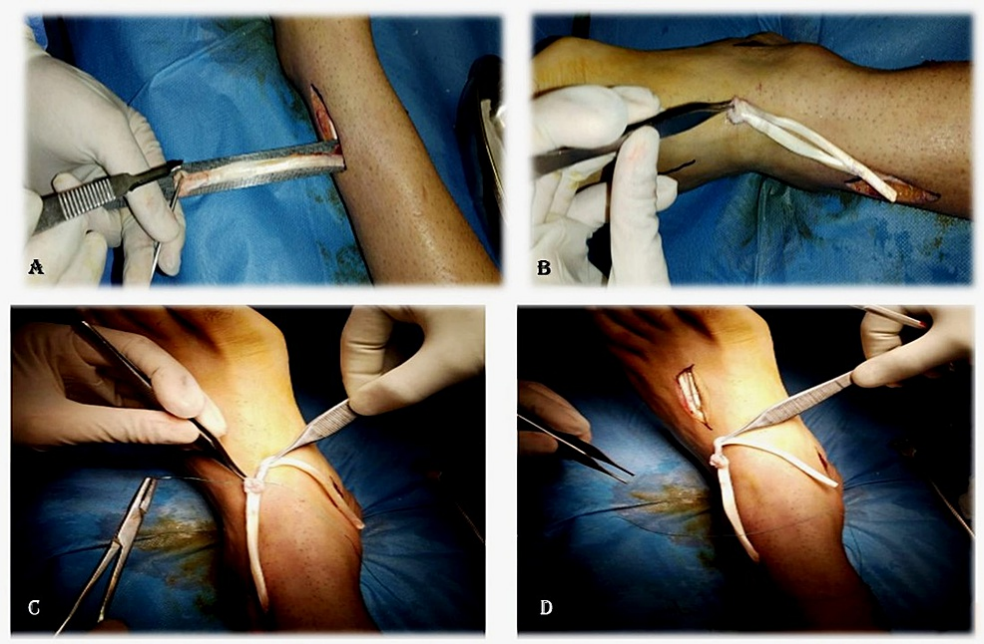
Lengthening of tibialis posterior tendon. Splitting and lengthening of tibialis posterior tendon (A, B). Securing of lengthened tibialis posterior tendon with non-absorbable suture (C, D).

**Figure 3:**
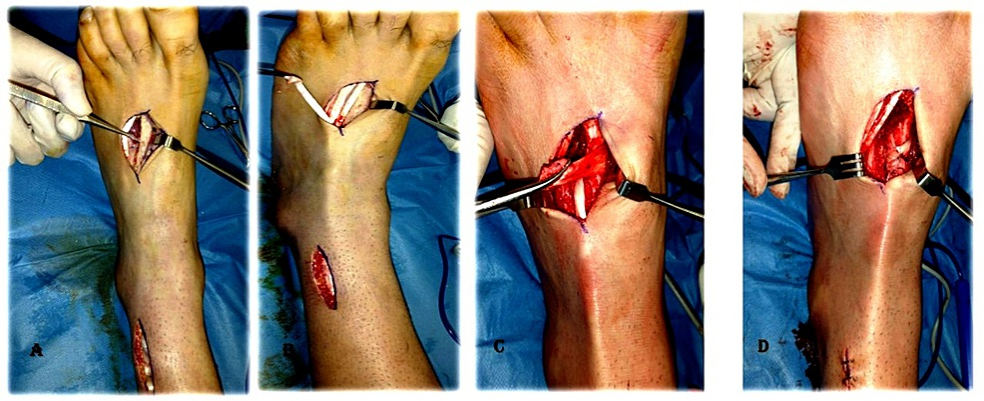
Fixation of tibialis posterior tendon to the 2nd metatarsal by looping around it. Second metatarsal bone exposed and denuded circumferentially at its proximal part (A). Delivery of lengthened tibialis posterior tendon to the foot dorsum through a subcutaneous tunnel (B). Looping of tibialis posterior tendon around the denuded 2nd metatarsal bone (C). Suturing of tibialis posterior tendon to itself after looping around 2nd metatarsal bone with non-absorbable suture material (D).

Passive range of motion of the ankle joint was assessed in all patients before surgery and in some patients with < 5o of dorsiflexion beyond neutral [1], “Z” lengthening of the Achilles tendon was performed. The tibialis posterior tendon was harvested and taken out to the foot dorsum via a subcutaneous route after passing through an interosseous tunnel (Figure 1). For tendon-to-tendon transfer, the tibialis posterior tendon was fixed to the tendon of tibialis anterior by pulvertaft weave method with non-absorbable suture while for tendon-to-bone transfer, when tibialis posterior tendon was brought to the lateral side of the leg after passing through the interosseous membrane, "L" lengthening of the TP tendon was performed to overcome the insufficient length. 2nd metatarsal was approached by a curvilinear incision, proximal part of the bone was denuded circumferentially, the TP tendon was passed around the bone and sutured to itself after making a loop around the 2nd metatarsal (Figures 2, 3). The ankle was kept at full dorsiflexion at the time of fixation in both techniques. For both the procedures, postoperative below-knee splintage was applied in dorsiflexion for six weeks, active dorsiflexion was initiated after six weeks and the patients were allowed to bear weight on the operated limb within the cast for the next six weeks while plantar flexion was not allowed during the whole 12-week duration. By the end of 12 weeks, the cast was removed and full weight-bearing was allowed.

From a total of 42, 38 patients fulfilled the inclusion criteria. The majority of them were operated on by tendon-to-bone fixation. The data was extracted from the registry regarding age, gender, limb involved, mechanism of injury, the time duration between injury and surgical intervention, type of procedure regarding the tendon attachment site, complication of surgery, and any additional surgery. The angle of active dorsiflexion after 6 months of surgery was also noted, which was been documented in the follow-up visit sheet of the patient in the registry

Ethical review was taken from the Ethical Review Committee of Liaquat National Hospital with ERC number App# 0564-2020 LNH - ERC. All patients were called and invited to participate in the study and to come for the follow-up visit in our outpatient department. 32 patients presented in the outpatient department for follow-up and were examined by a qualified plastic surgeon. The patients were divided into two groups according to the type of surgical technique acquired for fixation of tibialis posterior (TP) tendon to the foot dorsum, group 1- tibialis posterior to 2nd metatarsal (TP-2nd metatarsal), and group 2 - tibialis posterior to tibialis anterior tendon transfer (TP to TA). The range of active dorsiflexion was checked using a goniometer and categorized according to the scale presented by Carayon et al. (Table 1) [7,9]. The duration of surgery from the time of the last follow-up was also noted.

**Table 1:** The evaluation criteria of Carayon et al. The evaluation criteria of Carayon et al. [9] for the evaluation of patients who underwent tibialis posterior tendon.

Evaluation Criteria	Excellent	Good	Moderate	Poor
Active dorsiflexion	>15 degree	5-15 degree	No active dorsiflexion (no foot drop)	Presence of plantarflexion (foot drop)

Data were entered and analyzed through the statistical package for social sciences (SPSS) version25. The mean + SD were calculated for continuous variables i.e. age, the time duration between injury and surgery will be assessed. Frequency and percentages will be calculated for qualitative variables like gender, limb involved, mechanism of injury, the type of procedure used, and complications if any. The mean difference of angle of active dorsiflexion for both the techniques was compared by applying the Chi-square test (p-value >0.05). 

## Results

**Figure 4:**
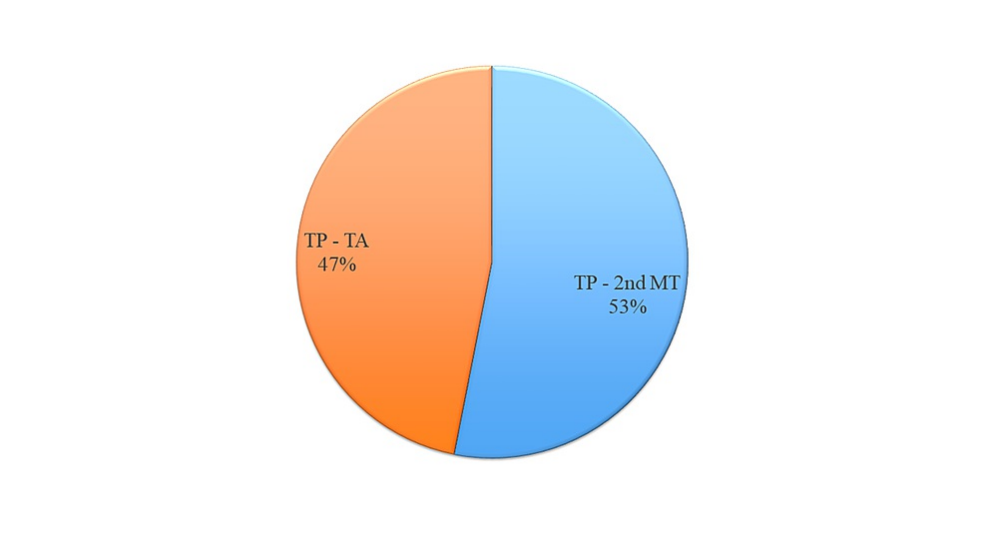
Procedure type (n = 32). Group 1: Tibialis posterior tendon was fixed to the second metatarsal bone. Group 2: Tibialis posterior tendon was fixed to the tibialis anterior tendon. TP: tibialis posterior; 2nd MT: second metatarsal; TA: tibialis anterior.

A total of 32 patients were included in the study (male 87.5%, female 12.5%) and were divided into two groups according to the type of surgical technique acquired for fixation of tibialis posterior (TP) tendon to the foot dorsum, group 1 (TP to 2nd metatarsal) n=17 and group 2 (TP to TA) n=15 (Figure 4).

The mean age of patients in group 1 was 28.35 + 6.13 years and in group 2 was 28.73 + 4.415 years (Table 2). Most of the patients in both groups were male and the mechanism of injury was penetrating trauma in the majority of the patients in both groups. No significant postoperative complications were noted in both the groups, one patient from each group developed a mild surgical site infection that was managed conservatively with antibiotics and dressings. Group-wise distribution of patients' gender, involved limb (right/ left), mechanism of injury, and complication is presented in Table 2.

**Table 2:** Group-wise distribution of demographic variables. TP: tibialis posterior; 2nd MT: second metatarsal; TA: tibialis anterior.

		Group 1 (TP-2nd MT)	Group 2 (TP-TA)
Age	Years (+SD)	28.35 (+6.13)	28.73 (+4.41)
Duration from injury to surgery	Mean (months + SD)	17.47 (+2.98)	19.13 (+7.51)
Gender	Male	15 (88.2%)	13 (86.7%)
	Female	2 (11.8%)	2 (13.3%)
Mechanism of injury	Blunt trauma	3 (17.6%)	1 (6.7%)
	Penetrating injury	11 (64.7%)	11 (73.3%)
	Gun Shot	2 (11.8%)	2 (13.3%)
	Other	1 (5.9%)	1 (6.7%)
Involved limb	Right	12 (70.6%)	7 (46.7%)
	Left	5 (29.5%)	8 (53.3%)
Complication	None	16 (94.1%)	14 (93.3%)
	Wound infection	1 (5.9%)	1 (6.7%)
	Wound dehiscence	0 (0.0%)	0 (0.0%)
	Other	0 (0.0%)	0 (0.0%)
Additional procedure	Yes	1 (5.9%)	1 (6.7%)
	No	16 (94.1%)	14 (93.3%)

**Figure 5:**
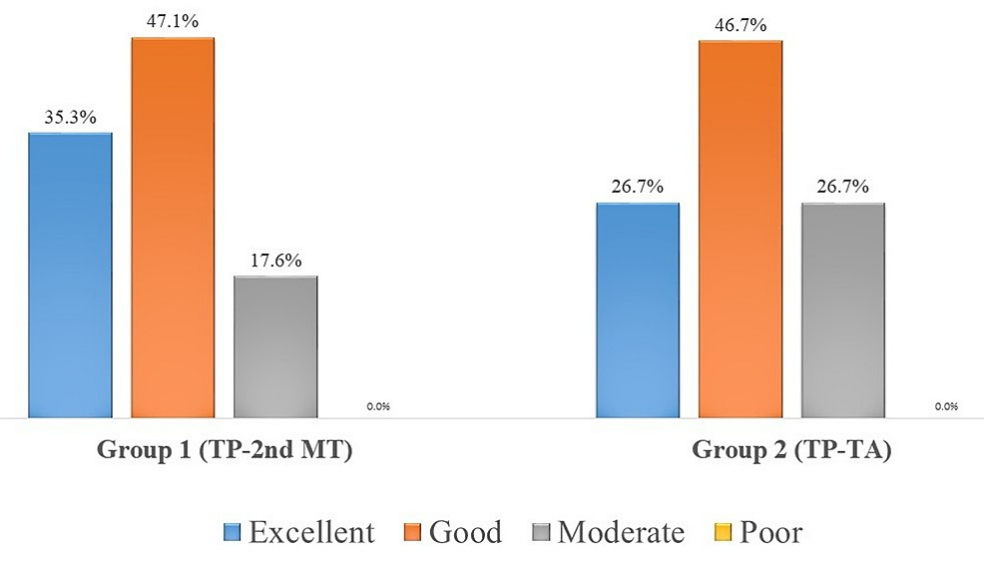
Six months post-operative: range of active dorsiflexion. Group 1: Tibialis posterior tendon was fixed to the second metatarsal bone. Group 2: Tibialis posterior tendon was fixed to the tibialis anterior tendon. Range of active dorsiflexion categorized as per Carayon et al. criteria. TP: tibialis posterior; 2nd MT: second metatarsal; TA: tibialis anterior.

**Figure 6:**
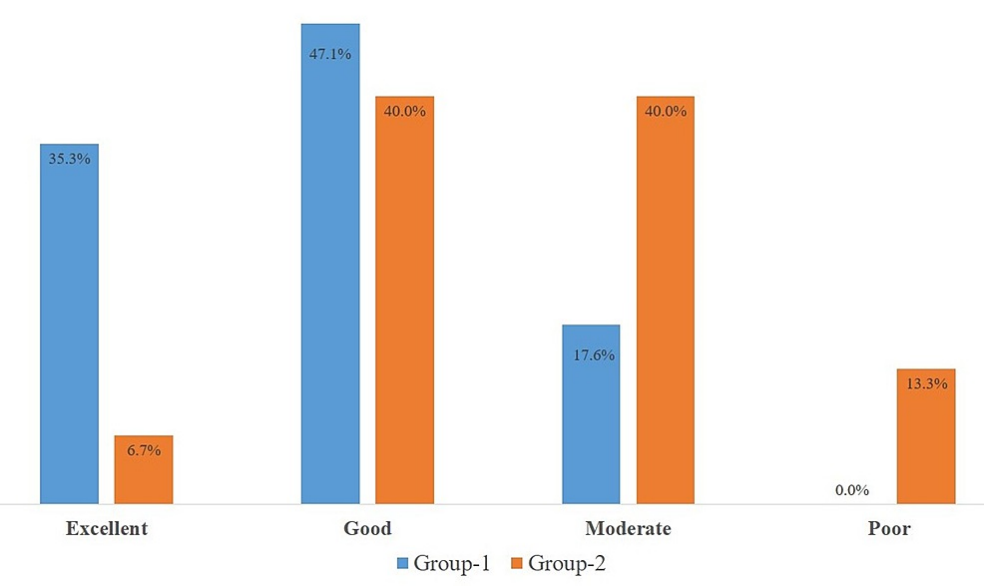
Mean 34.44 +10.11 months post-operative: active range of dorsiflexion. Group 1: Tibialis posterior tendon was fixed to the second metatarsal bone. Group 2: Tibialis posterior tendon was fixed to the tibialis anterior tendon. Range of active dorsiflexion categorized as per Carayon et al. criteria. TP: tibialis posterior; 2nd MT: second metatarsal; TA: tibialis anterior.

At 6 months postoperatively, the majority of the patients in both groups showed excellent to a good category as per the scale described by Carayon et al. (Figure 5). The mean follow-up time was 33.18 + 9.89 (23-59) months for group 1 and 35.87 + 10.54 (23-56) for group 2 patients at the time of final recordings and it was noted that in group-1 the results were excellent in 6 (35.3%), Good in 8 (47.1%) and moderate in 3 (17.6%), that is the same as after 6 months after the surgery while in group 2 patients it was excellent in 1 (6.7%), good in 6 (40.0%), moderate in 6 (40.0%) and poor in 2 (6.2%) patients, that might be due to stretching of the tendon over time (Figure 6). These results were compared using the chi-square test and it was statistically significant (p-value 0.016) which shows that insertion at the second metatarsal gives more favorable results as compared to insertion at tibialis anterior.

## Discussion

Drop foot has several aetiologies such as neurologic causes (peripheral nerve injury, neuropathy, lumbar radiculopathy, cerebral lesions) and muscular causes (extensor muscle injury, compartment syndrome). Out of these isolated peroneal nerve injury remains the most common cause of foot drop as it is more prone to injury due to its location. Some patients use ankle-foot orthosis (AFO) or brace to prevent foot drop but most of the patients can't tolerate it for a longer duration especially young ones [10]. A significant proportion of patients with peroneal nerve injury and subsequent foot drop require tendon transfer for restoration of normal toe-heel gait [10].

Tibialis posterior tendon transfer is the gold standard in the treatment of foot drop. Multiple sites have been suggested for the insertion of the tibialis posterior tendon on the dorsum of the foot [6]. We used mainly two different attachment sites on foot, one with a loop around the second metatarsal bone and the second one to the tibialis anterior tendon. The postoperative results of these two attachment sites were compared and the results were evaluated according to the scale described by Carlton et al. Total 32 patients were evaluated, 17 patients underwent tibialis posterior transfer with attachment on the second metatarsal and 15 on tibialis anterior tendon. Functional outcomes were assessed by grading of foot dorsiflexion at 6 months and 34.4 (mean) months.

At six months post-operative, the results were somewhat similar in both the groups approximately 80% of the patients had good or excellent results while after 34 months post-op, a significant difference in dorsiflexion was noted between the groups that showed deterioration of functional outcome in patients with tendon-to tendon (tibialis anterior) fixation i.e. 6.7% were excellent, 40% with good and 40% with moderate results, two of the patients from this group develop foot drop and started using ankle-foot orthoses (AFO).

Watkins et al., Codivilla [11], and Putti [12] are considered as the pioneers of the tibialis posterior tendon transfer to the dorsum of the foot through the interosseous route [13]. This technique has few drawbacks in that the length of the transposed tibialis posterior tendon was insufficient preventing tendon-to-bone fixation [13,14]. The novelty of our technique is that by lengthening of the tibialis posterior tendon before fixation to 2nd metatarsal, this issue has completely been addressed with maintaining sufficient strength of the tibialis posterior tendon with the restoration of balanced dorsiflexion and prevention of equinovarus deformity that can be caused by the unopposed pull of tibialis posterior tendon. No complication regarding slippage or rupture of the tibialis posterior tendon was noted. Tendon-to-bone fixation was done by Barr et al, who inserted the tendon to the intermediate or lateral cuneiform or base of 2nd metatarsal bone through the interosseous route [15]. Ober inserted the tendon to the base of the 3rd metatarsal through the circumtibial route [16]. In tendon-to-bone transfer, stable fixation was done by pull-out wire, staples, screws, or bone anchors [17]. Tendon-to-tendon fixation has been described in the literature as an alternative to tendon-to-bone transfer [18]. In direct tendon-to-tendon fixation, balanced dorsiflexion can't be achieved because the foot is pulled medially when the anterior tibialis tendon is used as a recipient [9,10,12].

Tendon-to-bone fixation is surgically demanding in terms of dissection and insertion of the tendon to the bone while the majority of the surgeons feel that tendon-to-tendon fixation is relatively easy to perform [18]. Another procedure was also mentioned in the literature in which tendon-to-tendon transfer was combined with tendon-to-bone fixation and showed that 81% of the patients developed active dorsiflexion beyond 0o, with a balanced foot posture [6].

For the treatment of permanent foot drop secondary to common peroneal nerve palsy, re-routing of tibialis posterior tendon through interosseous route and fixation to the 2nd metatarsal bone is a reliable method to restore the foot dorsiflexion and provides balanced and long term functional outcomes when compared with tibialis anterior tendon as a recipient. It provides the opportunity for the patients to walk with a normal gait without any need for ankle-foot orthosis (AFO). In future studies, further improvements can be made to enhance functional recovery and to improve the quality of life of the affected individuals. 

Our study was not out of limitations i.e. the sample size was small that can affect the generalizability of the results and there were aesthetic concerns that are multiple large scars on the leg and foot. In this transfer, we didn’t address the toes extension that after a certain period, may cause difficulty in using footwear.

## Conclusions

Tibialis posterior tendon transfer in permanent foot drop patients not only restores the active dorsiflexion but also prevents equinovarus deformity. Our modification provides adequate length for looping around and fixation of the transferred tibialis posterior tendon at the second metatarsal. The tendon-to-bone (2nd metatarsal) gives more favorable results and provides balanced dorsiflexion as compare to tendon-to-tendon (tibialis anterior) transfer as the quality of active dorsiflexion deteriorates over time probably due to stretching of the tendon. In cases where active dorsiflexion wasn't good enough, the tendon-to-bone fixation acts as an internal splint and prevents the foot drop alleviating the need for AFO.
